# Supplementary material for: Expression of Low Level of VPS35-mCherry Fusion Protein Diminishes Vps35 Depletion Induced Neuron Terminal Differentiation Deficits and Neurodegenerative Pathology, and Prevents Neonatal Death
Source: Int J Mol Sci. 2021 Aug 4;22(16):8394. doi: 10.3390/ijms22168394 (PMC8395035; doi:10.3390/ijms22168394)
Supplement: Supplementary file 1 [file ijms-22-08394-s001.zip › ijms-1256282-supplementary/Supplementary files/Supplementary fig legends.pdf]

Fig S1. Generation of Tg*Vps35*^{Neurod6} mice. **(A)** Illustration of generation of LSL-Vps35-mCherry and LSL-Vps35-mCherry; Neurod6-Cre mice. **(B)** Western blot analysis of Vps35 and Vps35-mCherry levels in HEK-293 cells transfected with the indicated plasmids. **(C)** Immunostaining of HEK-293 cells transfected with the indicated plasmids. **(D)** Western blot analysis of Vps35-mCherry levels in different brain tissues taken from control (*Vps35*$^{f/f}$) and *TgVps35*^{Neurod6} mice, β-actin was employed as a loading control. **(E)** Relative quantitation of the protein levels reveals the specific expression of mCherry in cortex and hippocampus, not cerebellum. **(F)** Body weight curves of *TgVps35*^{Neurod6} mice and littermate controls. **(G)** Representative Nissl stains of control and *TgVps35*^{Neurod6} mice at indicated age. **(H)** Quantification analysis of Nissl stains that revealed a comparable cortical thickness between control and *TgVps35*^{Neurod6} mice (n = 3~4 animals per genotype, take 8-10 positions for each animal; two-tailed unpaired t test). **(I, J)** Representative images of immunostaining analysis using indicated antibodies in P14 neocortical and hippocampal sections from control and *TgVps35*^{Neurod6} mice. Higher magnification images of the boxed regions were shown in offside and lower panels. **(K, M)** Representative images of immunostaining analysis using indicated antibodies in P14 neocortical sections from *TgVps35*^{Neurod6} mice. **(L, N)** Quantification analysis of co-immunofluorescence from K and M. Scale bars as indicated in each panel. Individual data points were shown as dots with group mean ± s.e.m; ** $p < 0.01$; *** $p < 0.001$; n.s., not significant.

Fig S2. Terminal differentiation deficits of *TgVps35*^{Neurod6}*; KO* mice after P14. **(A)** Schematic drawing showing the process of AAV-Syn-mCherry injection. **(B)** Representative images of single neuron labeled with mCherry from hippocampus CA1 and entorhinal cortex. **(C)** Quantification of dendritic complexity and total dendritic length of hippocampal CA1 pyramidal neurons from control, *Vps35*^{Neurod6} and *TgVps35*^{Neurod6}*; KO* mice at P29 (n=3 neurons from 3 mice per genotype; one-way ANOVA with Tukey's multiple-comparison test). **(D)** Quantification of dendritic complexity and total dendritic length of entorhinal cortex pyramidal neurons from control, *Vps35*^{Neurod6} and *TgVps35*^{Neurod6}*; KO* mice at P29 (n=3 neurons from 3 mice per

genotype; one-way ANOVA with Tukey's multiple-comparison test). Scale bars as indicated in each panel. Individual data points were shown as dots with group mean ± s.e.m; * $p < 0.05$; ** $p < 0.01$; *** $p < 0.001$; **** $p < 0.0001$; n.s., not significant.
