# Supplementary figures and images for: Expression of Low Level of VPS35-mCherry Fusion Protein Diminishes Vps35 Depletion Induced Neuron Terminal Differentiation Deficits and Neurodegenerative Pathology, and Prevents Neonatal Death
Source: Int J Mol Sci. 2021 Aug 4;22(16):8394. doi: 10.3390/ijms22168394 (PMC8395035; doi:10.3390/ijms22168394)

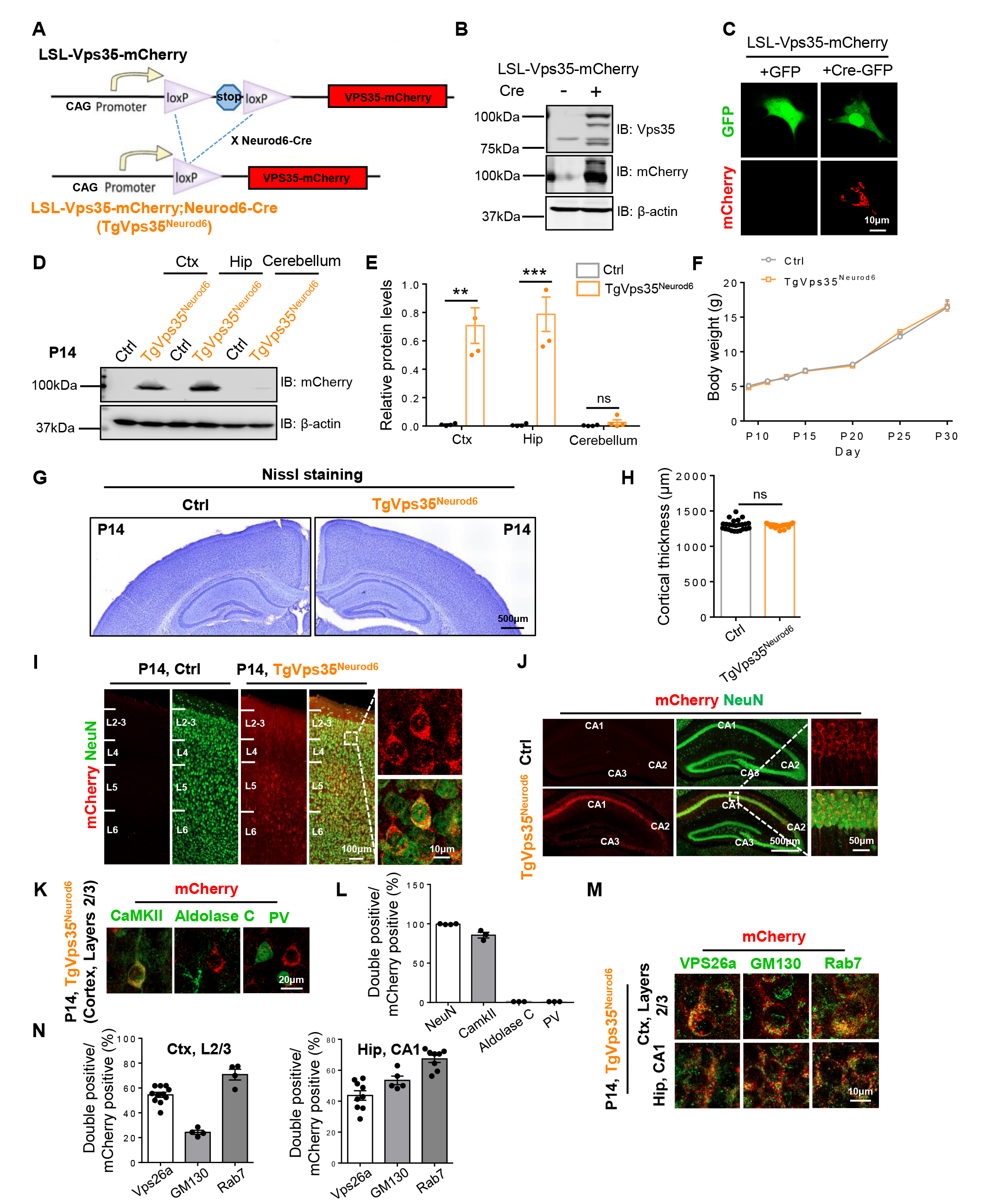

Supplement: Supplementary file 1 [file ijms-22-08394-s001.zip › ijms-1256282-supplementary/Supplementary files/Fig S1.tif]

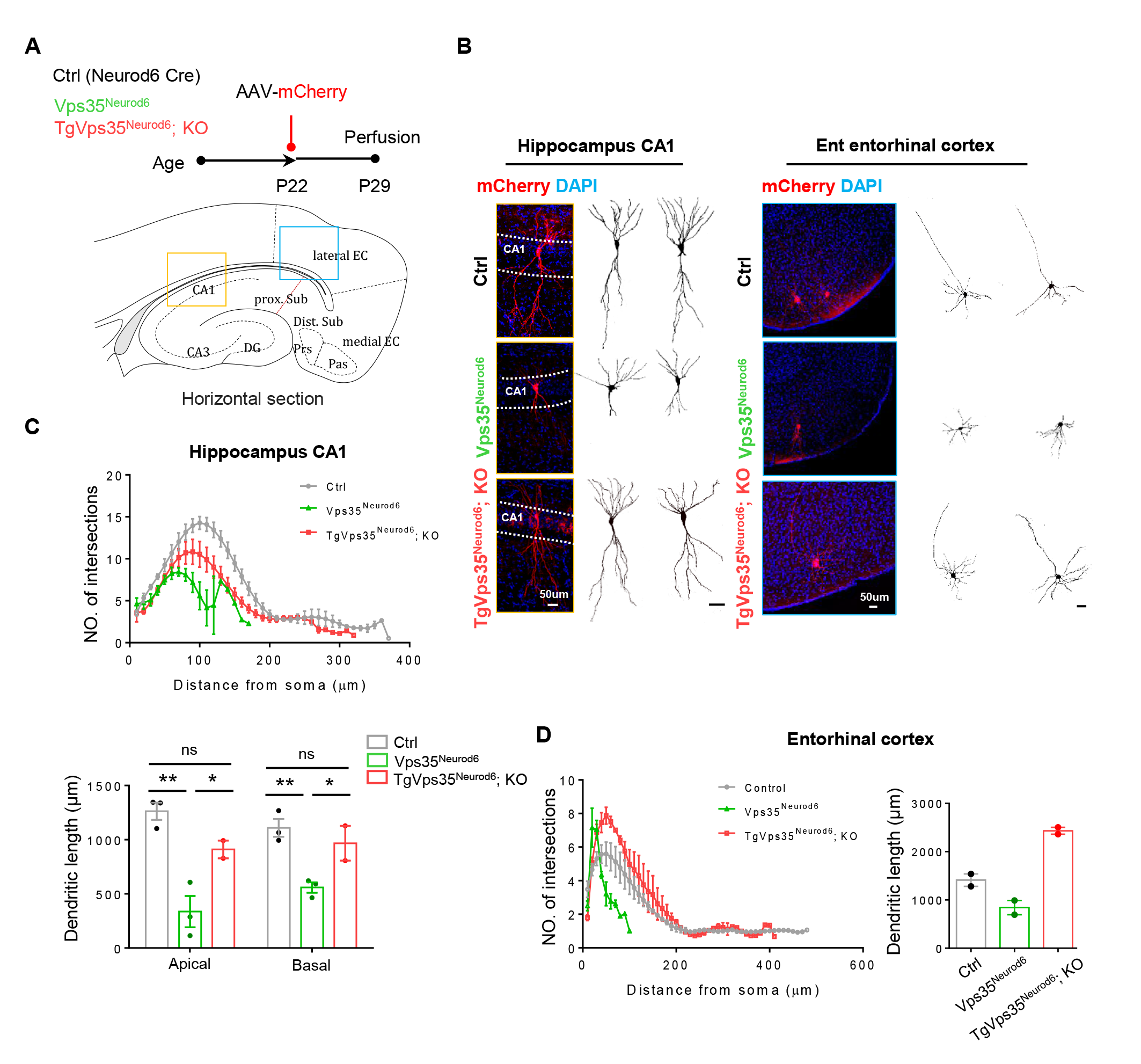

Supplement: Supplementary file 1 [file ijms-22-08394-s001.zip › ijms-1256282-supplementary/Supplementary files/Fig S2.tif]
